# Calcifying Nested Stromal-Epithelial Tumor: An Extremely Rare Hepatic Tumor

**DOI:** 10.14309/crj.0000000000001163

**Published:** 2023-10-03

**Authors:** Bilal Ahmed Khan, Imran Ali Syed, Ihsan Ulhaq, Sohail Rashid, Muhammad Yasir Khan, Abdullah Khalid, Urfa Shafi, Usman Iqbal Aujla, Faisal Saud Dar

**Affiliations:** 1 Pakistan Kidney and Liver Institute & Research Center, DHA Phase VI, Lahore, Pakistan

**Keywords:** calcifying nested stromal-epithelial tumor, spindle cells, epithelioid cells

## Abstract

Calcifying nested stromal-epithelial tumor is a rare hepatic malignancy with approximately 50 cases reported in the literature. Its clinical presentation is nonspecific, and the diagnosis is mainly based on histology which shows nests of spindle and epithelioid cells along with a desmoplastic myofibroblastic stroma containing variable calcification and ossification. In this report, we present a case of a 24-year-old woman with a history of abdominal pain, distension, and dyspepsia. She had a palpable liver with normal liver function test results. Serum alpha-fetoprotein levels were within normal range, and serologies for hepatitis B and C virus remained negative. Radiological investigations (magnetic resonance imaging and computed tomography) showed a large, right hepatic lobe mass with tumor invasion into the right posterior portal vein, but the 2 modalities could not characterize the lesion. Finally, an ultrasound-guided biopsy of the liver lesion provided the diagnosis of calcifying nested stromal-epithelial tumor. The tumor was resected successfully.

## INTRODUCTION

Calcifying nested stromal-epithelial tumor (CNSET) is a rare hepatic malignancy. The tumor was first described by Ishak et al in 2001. Since then, approximately 50 cases have been reported in the literature either as individual case reports or as small case series.^1^ The histogenesis of this tumor remains unknown, but histologically, it has been described as a nonbiliary, nonhepatocytic tumor of the liver composed of nests of spindle and epithelioid cells along with a desmoplastic myofibroblastic stroma containing variable calcification and sometimes ossification as well.^2^

Clinically, it may present with abdominal pain, a mass, ileus, or as an incidental finding in an asymptomatic patient. Various associations of CNSETs have been described in the literature, such as cortisol-related syndrome and Beckwith-Wiedemann syndrome. The tumor is more common among young children and female patients.^2–4^ In this report, we present a patient who underwent right hepatectomy for a large CNSET.

## CASE REPORT

A 24-year-old woman presented with several months’ history of constant right hypochondriac pain, which was nonradiating. Initially, its intensity was mild, which progressed to moderate over a period of 3 weeks. The pain used to relieve temporarily with analgesics. It was associated with abdominal fullness and bothersome dyspepsia. She denied the use of oral contraceptive pills or any herbal medication. Her medical and surgical history was insignificant. Her physical examination was unremarkable, except for a palpable liver border below the right costal margin.

Laboratory investigations revealed normal liver function tests (aspartate aminotransferase 27 IU/L, alanine aminotransferase 24 IU/L, alkaline phosphatase 97, and gamma-glutamyl transferase 40 IU/L). Serum alpha-fetoprotein was normal, and virological markers for hepatitis B and C yielded negative results.

**Figure 1.**
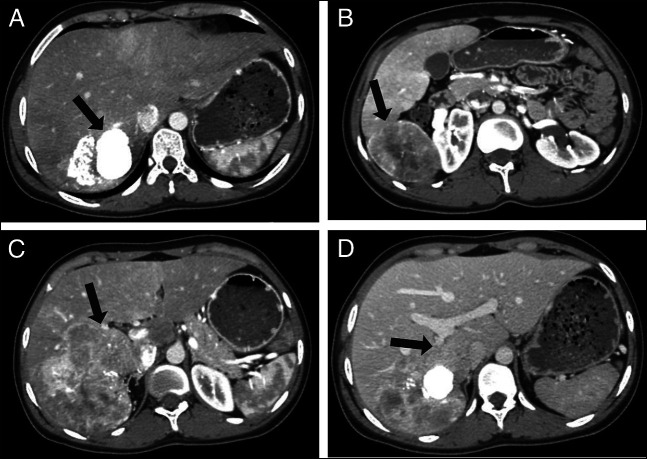
Abdominal MRI with contrast. (A, B) T1-weighted axial sections showing a large CNSET (white arrow) involving the right lobe of the liver. (C) T2-weighted axial section showing the tumor with a central scar. (D) T2-weighted image showing an exophytic component of the tumor on the coronal section. CNSET, calcifying nested stromal-epithelial tumor; MRI, magnetic resonance imaging.

**Figure 2.**
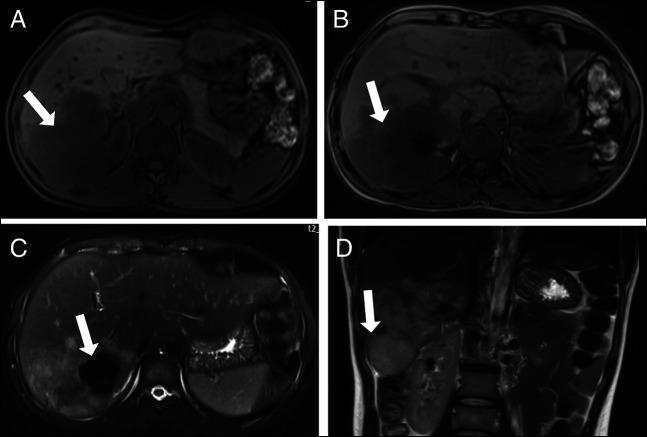
Contrast-enhanced CT scan of the abdomen. (A) Axial section showing a large CNSET with central calcification (black arrow). (B) Axial section showing a exophytic component of the tumor (black arrow). (C) Axial section showing the involvement of a major portion of the right hepatic lobe (black arrow). (D) Axial section showing tumor invasion into the right posterior portal vein (black arrow). CNSET, calcifying nested stromal-epithelial tumor; CT, computed tomography.

Initially, a triphasic liver dynamic computed tomography scan was performed, showing a smoothly marginated liver with a fairly well-defined right lobar lesion based on segments V, VI, and VII having an inferior exophytic component with central coarse chunky intralesional calcifications with no convincing washout on venous and delayed phases along with associated tumor thrombus in the right posterior portal vein (Figure 1). Subsequently, to characterize the lesion, abdominal magnetic resonance imaging was performed, revealing a solid lesion with a cystic component in segments VI and VII measuring 9.8 × 9.4 × 12 cm. It had a heterogeneous arterial phase enhancement that persisted through the venous and delayed phases (Figure 2). The major differential diagnoses based on the abovementioned radiologic findings included cholangiocarcinoma, atypical hepatocellular carcinoma, neuroendocrine tumor, or hepatic adenoma. Hence, it was decided to perform ultrasound-guided biopsy of the hepatic lesion for tissue diagnosis.

**Figure 3.**
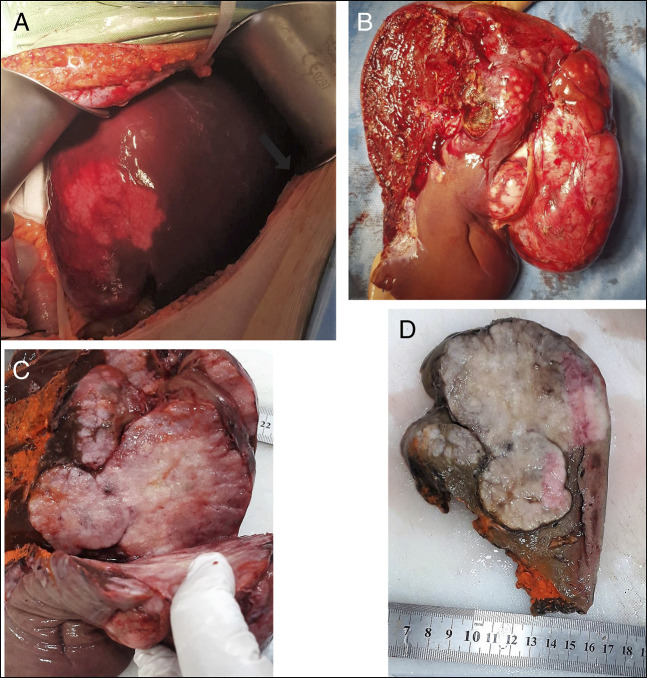
Gross appearance of the CNSET. (A) Per operative appearance of the tumor. (B) Surgically resected right lobe of the liver with a large CNSET having an exophytic component. (C, D) Cut surface of the tumor with a lobulated gray and white appearance and calcification, whereas the resection margin painted orange is free of tumor. CNSET, calcifying nested stromal-epithelial tumor.

The histopathological findings (described later in the report) were consistent with the diagnosis of a CNSET of the liver. The case was discussed in the multidisciplinary team meeting, and surgical resection of the tumor was recommended. A laparotomy was performed, revealing a CNSET arising from the segments V, VI, and VII of the right liver lobe, measuring approximately 10 × 12 cm (Figure 3). The remaining liver was normal in appearance with an adequately sized left hepatic lobe. Right hepatectomy was performed, and the transection plane was kept on the right side of the middle hepatic vein. The resected liver specimen was sent for histopathological examination. The gross examination showed a resected liver specimen measuring 15 × 15 × 10 cm with an exophytic tumor component measuring 13 × 9.0 × 8.0 cm. It was 1.5 cm away from the parenchymal resection margin. On serial sectioning, the tumor appeared well-circumscribed and had a lobulated appearance with extensive calcifications (Figure 3).

**Figure 4.**
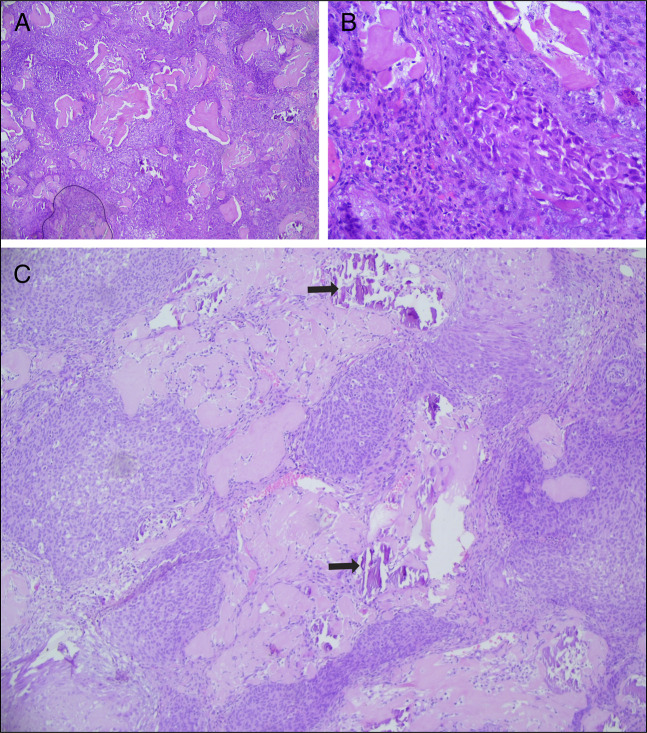
Histopathological appearance of CNSET. (A) Sheets and nests of tumor cells with osteoid formation (100× magnification). (B) Sheets and nests of spindle and epithelial cells with uniform nuclei (400× magnification). (C) Tumor with osteoid formation and calcification (black arrow) at 100× magnification. CNSET, calcifying nested stromal-epithelial tumor.

**Figure 5.**
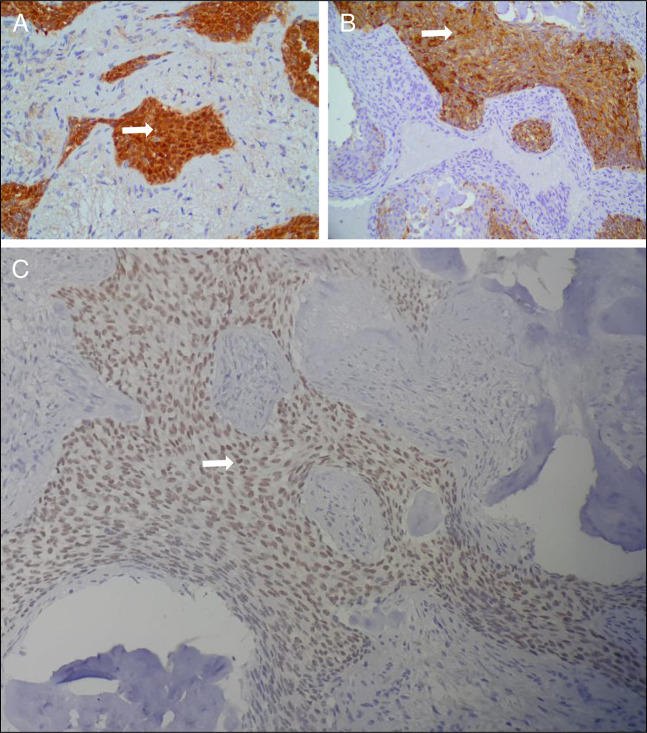
Immunohistochemistry images of CNSET. (A) Nuclear positivity for beta-catenin (white arrow) in neoplastic cells at 100× magnification. (B) Membranous positivity for cytokeratin AE1/AE3 (white arrow) at 100× magnification. (C) Nuclear positivity for WT1 (white arrow) at 100× magnification. CNSET, calcifying nested stromal-epithelial tumor.

Microscopic examination of the involved liver tissue showed a neoplasm composed of nests of spindled to epithelioid cells with uniform nuclei separated by a fibrous stroma. There was extensive osteoid deposition accompanied by calcification. Cords of benign hepatocytes and bile ducts were also seen entrapped within the stroma. There was no necrosis or marked nuclear atypia. Beta-catenin (both nuclear/membranous staining) and Wilms tumor antibody were positive; cytokeratin, glypican3, and Hep-Par1 were negative (Figures 4 and 5). The postoperative course remained uneventful, and the patient was discharged from the hospital after 1 week in a stable condition.

## DISCUSSION

CNSET is a primary, but rare, hepatic malignancy. Makhlouf et al^5^ proposed the name CNSET. It has been described in the literature with various names, including desmoplastic nested spindle cell tumor, ossifying stromal-epithelial tumor, and ossifying malignant mixed epithelial and stromal tumor.^6^ The histogenesis of this tumor is still unknown. One hypothesis is that CNSET is derived from hepatic mesenchymal precursor cells with differentiation along the bile duct lineage. Another hypothesis is that it might develop from calcified benign liver lesions. Some cases of CNSET have been reported in the literature who had a history of calcified liver lesions, which were believed to be hepatic hemangiomas, later turned into CNSET.^7^

CNSET exhibits a broad range of presentations. The literature review showed that its clinical presentation is nonspecific and varies from incidental findings with no symptoms to a variety of symptoms, including abdominal pain, distension, dyspepsia, weight gain, and, occasionally, jaundice and fever. In most of the reported cases, tumors were found incidentally.^8^ What sets this patient's presentation apart is the culmination of symptoms, such as abdominal pain, distension, and dyspepsia. Notably, associations with conditions such as ectopic production of adrenocorticotropic hormone culminating in Cushing syndrome or with Beckwith-Wiedemann syndrome, identified in some cases, were conspicuously absent in our case report.

Diagnosis of CNSET mainly relies on radiological imaging and histopathological findings. There are no specific tumor biomarkers for CNSET. Liver function tests, alpha-fetoprotein, and carcinoembryonic antigen levels remain within the normal range in most cases. Treatment options include surgical resection, liver transplantation, and radiofrequency ablation. Chemotherapy (hepatoblastoma or soft-tissue sarcoma protocol-based) has also been reported in the literature.^2,9^

Most of the reported cases had low-grade histology and followed an indolent clinical course. To the best of our knowledge, among the 50 reported cases, only 8 had a recurrence and 5 cases showed distant metastasis.^9^ No disease recurrence or metastasis was observed in our case report at the 1-year follow-up.

CNSETs affect the female sex predominantly. Histopathological examination plays a key role in its diagnosis. There are no standard guidelines available for its management. At present, surgical management for liver resection and transplantation are the mainstay of treatment. Further studies on pathogenesis and histogenesis are required to devise more accurate diagnostic tools and discover effective treatment strategies.

## DISCLOSURES

Author contributions: BA Khan: conception, manuscript revision, and literature review. IA Syed: manuscript writing and revision and is the article guarantor. I. Ulhaq, S. Rashid, MY Khan, and A. Khalid: manuscript revision and edition for intellectual content. U. Shafi: provided histopathological images and described the findings. UI Aujla: revision, manuscript writing, and critical analysis. FS Dar: critical analysis and final approval.

Financial disclosure: None to report.

Informed consent was obtained for this case report.
